# Divergent approaches in the vaccination of recently arrived migrants to Europe: a survey of national experts from 32 countries, 2017

**DOI:** 10.2807/1560-7917.ES.2018.23.41.1700772

**Published:** 2018-10-11

**Authors:** Sally Hargreaves, Laura B Nellums, Sofanne J Ravensbergen, Jon S Friedland, Ymkje Stienstra, on behalf of the ESGITM Working Group on Vaccination in Migrants

**Affiliations:** 1 These authors are joint first authors; 2 The Institute for Infection and Immunity, St George’s, University of London, London, United Kingdom; 3 The International Health Unit, Section of Infectious Diseases and Immunity, Imperial College London, London, United Kingdom; 4 Department of Internal Medicine/Infectious Diseases, University of Groningen, University Medical Centre Groningen, Groningen, the Netherlands

**Keywords:** vaccination, immunisation, migrants, European Union, public health policy, travel

## Abstract

Migrants within the European Union and European Economic Area (EU/EEA) may be underimmunised and lack documentation on previous vaccinations. We investigated approaches to vaccination in recently arrived adult and child migrants, and guideline availability and implementation. **Methods**: Between March and May 2017, a national vaccination expert from every EU/EEA country and Switzerland completed an electronic questionnaire. We used descriptive analyses to calculate percentages, and framework analysis to synthesise free-text responses. **Results**: We approached 32 countries (response rate 100%). Although 28 experts reported vaccination guidance at national level, specific guidelines for recently arrived migrants were only available in six countries and not consistently implemented. Twenty-three countries administered vaccinations during on-arrival health checks. Most experts recommended multiple vaccination opportunities be made available: at point of entry (n = 13) or at holding level (reception centres, migrant camps, detention centres) (n = 21). In 30 countries, child migrants without evidence of previous vaccination were re-vaccinated according to the national schedule. Diphtheria-pertussis-tetanus and polio vaccinations were given to migrant children in all countries, measles-mumps-rubella (MMR) in 31 countries, hepatitis B vaccination in 25. Low levels of catch-up vaccination were reported in adult migrants, with only 13 countries offering MMR and 10 countries charging fees. **Conclusion**: Existing guidance is often not migrant-specific and may not be applied in practice; clarification is needed on which vaccines should be given. Strategies are needed specifically for catch-up vaccination in adult migrants. Vaccinations should be offered in multiple settings, free of charge, with sufficient guidance and training provided to front-line healthcare professionals.

## Introduction

Ensuring high levels of vaccination coverage is a key priority for the European Union (EU) [1-4]; yet very high levels of both external and internal migration in the region in recent years have posed considerable challenges to achieving this. Migrants, including refugees and asylum seekers, may be underimmunised if they have come from countries whose healthcare system has been disrupted due to war or other circumstances, which makes them vulnerable to acquiring infection if exposed [5-7]. Syrian and Afghan migrants, dominant migrant groups to the EU in recent years [8], have relatively low vaccine coverage rates. For example, immunisation coverage in Syria is around 40% for diphtheria, tetanus and pertussis (DTP) and 50% for polio [9,10]. Greece recently reported vaccination status as ‘unknown’ in 79.3% of Syrian children during an outbreak of hepatitis A in migrant camps [11]. Outbreaks of vaccine-preventable diseases such as measles have been seen among migrants in Europe, which may reflect sub-optimal vaccination coverage in migrant populations [12,13]. Many migrants lack any documentation of their vaccination history. The role of serology in assessing vaccination status is not clear and clinically relevant information about the usefulness of serology for migrants arriving in host countries is not available. Serological testing is, for example, not recommended for polio in migrants arriving to the United States, [14], but it is used for other infections in other groups, for example travellers going abroad and presenting for pre-travel advice. Most countries do not routinely check serology before vaccination of arriving migrants because of cost and logistical issues.

On arrival to the receiving country, migrants may face multiple barriers to accessing healthcare, including catch-up vaccinations [15,16]. Migrants are known to face barriers to accessing primary-care physicians, where most vaccination and screening for infection routinely occurs [17], and may be charged for any healthcare they receive, which may mean that seeking preventative healthcare such as vaccination becomes less of a priority [18]. Undocumented migrants in particular may fear approaching health services because of links with immigration authorities.

The European Vaccine Action Plan (EVAP) has set out a series of goals and objectives for immunisation and control of vaccine-preventable diseases (VPD) in the European Region member states for 2015–20 [4], emphasising that special attention should be paid to migrants and marginalised communities, ensuring their eligibility and access to appropriate immunisation services and information. However, strategies and approaches to engaging this group are lacking, as are high-quality studies assessing vaccination implementation in migrant populations [19]. The Promote Vaccination among Migrant Populations in Europe (PROVOMAX) project, which ended in 2013, sought to promote vaccination among migrants and develop recommendations for policymakers [20]. The European Centre for Disease Prevention and Control (ECDC) Vaccine Scheduler database (https://vaccine-schedule.ecdc.europa.eu) highlights immunisation schedules in all EU countries and allows comparison of vaccination policies between countries [21]. This provides a sense of what every country is doing, but the database does not have data on vaccine schedules for migrants.

There remain numerous questions around optimal vaccination strategies in migrants, including which vaccinations should be prioritised in adult and child migrants and how to promote vaccination uptake in this group and implement effective and cost-effective programmes. We therefore approached national vaccination experts from every EU and European Economic Area (EEA) country and Switzerland to complete an electronic questionnaire survey exploring current and preferred approaches to vaccination in recently arrived migrants, guideline availability and implementation, different approaches in adults and children, the extent to which charges or fees were applied. The survey also contained open-ended questions to allow experts to document promotional activities in migrants and perspectives from across the region.

## Methods

### Questionnaire development

We developed an electronic 12-point questionnaire survey containing structured and open-ended questions around country-specific vaccination policies for recently arrived migrants in the EU/EEA and Switzerland. Switzerland was included because the country has been hosting large numbers of refugees since 2015. This approach of engaging national experts has been successfully used previously in this field [22]. Questionnaire development was informed by a narrative synthesis of existing literature on migrant vaccination in Europe. For the purposes of this research, we defined recently arrived migrants as foreign-born and living in the host country for less than 10 years. At the top of the questionnaire we alerted experts to the fact that recently arrived migrants included a variety of migrants, specifying definitions for refugees (granted asylum in the host country), asylum seekers (awaiting a decision on their asylum application) and undocumented migrants (without the necessary authorisation or documents required under the host country’s immigration regulations). We defined children as individuals aged between 0 and 18 years.

The questionnaire (Supplement 1) included specific questions on the availability of national or regional guidelines for vaccinations in recently arrived migrants, and the extent to which they are applied in practice. In addition, questions were asked about what vaccinations are currently given, differences between adults and children, and the experts’ opinions on new approaches, where and what should be offered, approaches adopted for migrants with incomplete vaccination history or lack of documentation, and whether migrants are charged a fee for vaccinations received. In the open-ended questions, we asked experts to provide specific examples of innovative strategies and promotional activities around vaccination and immunisation currently aimed at recently arrived migrants in their countries. The questionnaire was designed to take around 15 min to complete.

### Approach and data analysis

Before distributing the survey, we piloted it with two members of the European Society of Clinical Microbiology and Infectious Diseases (ESCMID) Study Group for Infections in Travellers and Migrants (ESGITM). These two interviews were excluded from the analysis but were used to improve the questions and instructions. We amended the questionnaire based on their feedback. We created the ESGITM Working Group on Vaccination of Migrants, a group of European experts on infection and vaccination, and all members of the working group were asked to recommend a vaccination expert in their country. These vaccination experts had to be working at a national level (e.g. the Ministry of Health, a public health institution or equivalent) with expertise relating to vaccination policy and practice in migrants in their specific country. For six countries, for which a recommendation was not given by the ESGITM network, experts were identified through a search of authors of national guidelines and vaccinations documents for that specific country. These experts were contacted and asked whether they could complete the survey for their country or recommend another expert. We aimed to approach one national expert from each country. The questionnaire was sent electronically via email between March and May 2017 to experts in the following countries: Austria, Belgium, Bulgaria, Croatia, Cyprus, Czech Republic, Denmark, Estonia, Finland, France, Germany, Greece, Hungary, Iceland, Ireland, Italy, Latvia, Liechtenstein, Lithuania, Luxembourg, Malta, the Netherlands, Norway, Poland, Portugal, Romania, Slovakia, Slovenia, Spain, Sweden, Switzerland and the United Kingdom. A first reminder was sent after 2 weeks. A second reminder was sent 1 month later. Experts were asked to complete the electronic form and email it back to us.

Data were extracted from the completed survey forms by two researchers, and inputted into Microsoft Excel, to ensure accuracy before analysis. Descriptive analyses were conducted to calculate percentages and proportions. Framework analysis was conducted to synthesise free-text responses in the open-ended questions [23].

## Results

### Survey response

All 32 experts from the 32 approached EU/EEA countries and Switzerland returned a completed questionnaire. Eight were working in Ministry of Health teams specifically on migration, 21 in public health teams, three had expertise in vaccination issues related to migrants – for example being part of vaccination advisory groups (18 women, 14 men). Detailed information on the expert group and their expertise can be found in Supplement 2.

### Vaccination guidelines: policy vs practice

Twenty-eight of 32 experts reported being aware of guidance at a national level on vaccination within their country, yet guidelines specifically focusing on migrants were only reported by six of 32 experts. Twenty-three experts reported that vaccinations were administered during an on-arrival health check to recently arrived migrants, 29 experts reported that recently arrived migrants were offered a health check within a month after arrival, and in 17 countries, this health check was compulsory. Countries followed the national schedule when seeing a migrant for the first time, with 14 of 32 experts stating that national vaccination guidelines were always applied in practice in migrant patients. Sixteen experts reported that the guidelines were only partly applied in practice, whereas two reported that guidelines were never applied in practice. Experts reported that the extent to which national guidelines (or where available, migrant-specific guidelines), were implemented depended on the number of healthcare staff available, the number of refugees, willingness of healthcare staff and awareness among healthcare staff as to the immunisation needs of presenting migrants.

### Differences in vaccination approach between children and adults

**Figure 1:**
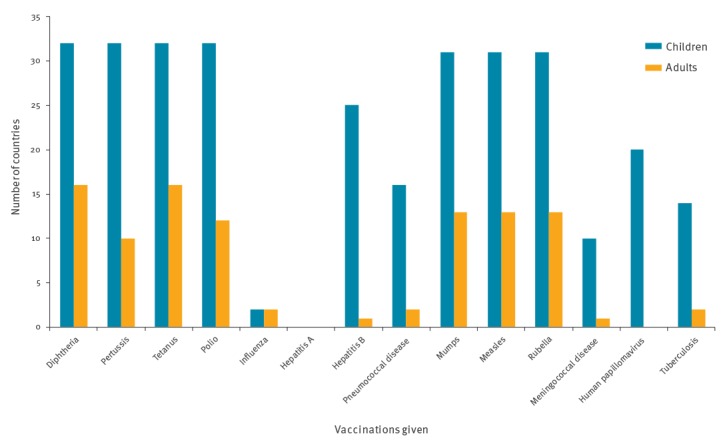
Vaccinations administered to adult and child migrants: approaches identified across Europe, 2017 (n = 32 countries)

The vaccines offered to adults and children varied across countries, according to the experts consulted (Figure 1). DTP, polio and measles-mumps-rubella (MMR) vaccinations were given to migrant children in 31 of 32 countries, with hepatitis B vaccination being the next most commonly administered vaccine (25/32). In half or less than half of all reporting countries were child migrants offered vaccinations against tuberculosis, meningococcal disease, pneumococcal disease or influenza. Recently arrived adult or child migrants were not vaccinated for hepatitis A in any country. Adult migrants seem to be excluded from catch-up vaccination initiatives in most countries, with experts reporting lower numbers of different vaccinations per person. Adult vaccination mainly focused on catch-up vaccinations for DTP, polio and MMR, but half or less than half of all reporting countries reported offering these vaccinations to adults (diphtheria: 16/32; pertussis: 10/32; tetanus: 16/32; polio: 12/32; MMR: 13/32). Other vaccines were less frequently reported. Data were not collected in this survey on the approach taken when multiple doses of a vaccination are required.

### Approaches adopted for migrants reporting incomplete vaccination history

The approach in migrants with an incomplete vaccination history or a lack of documentation varied by country. In most countries, when there was a lack of evidence of previous vaccination in children, or evidence of incomplete vaccination, they were re-vaccinated according to the national schedule (n = 30). For adults, four countries experts reported that adults in this situation would not be vaccinated for anything. In 18 countries, vaccinations that are prioritised in order to prevent an outbreak were administered to adults, whereas re-vaccination in children occurred according to the national vaccination schedule. One of the 32 experts reported that they would do serological testing before repeat vaccinations in adults.

### Financing of vaccination for migrants in the EU/EEA

Ten experts reported that migrants had to pay for their vaccination when approaching statutory services. In three of these 10 countries, experts stated that it was specifically undocumented migrants that had to pay for vaccinations. The need for financial contribution also varied by age; one expert reported that vaccination in children was free of charge and that only adults were charged. Another expert reported that vaccination upon arrival at the first medical check was always offered for free, whereas vaccinations at a later stage had to be paid by certain migrants.

### Promotional activities and innovative strategies

Fifteen experts reported initiatives to engage migrants in vaccination and improve uptake, some examples of which are outlined in Table 1. Promotional activities and innovative strategies were organised at different levels of the healthcare system; Table 1 shows the diversity of the strategies across Europe. However, we have no data on how effective or evidence-based these different approaches are, and where leaflets had been translated to make them more accessible they were not always translated into sufficient dominant migrant languages. In addition, it needs to be acknowledged that a lack of literacy in some migrant groups can be a major barrier to healthcare and vaccination.

**Table 1:** Strategies for improving vaccination uptake in adult and child migrants, reported by vaccine experts in the EU/EEA, 2017 (n = 32 countries)

Theme	Examples of activity and strategy
Distribution of promotional material	*Peer-to-peer projects* Sweden: Peer-to-peer project combined with a package of communication material (film, web-based animation, dialogue seminars) in the Somali community based on the Tailoring Immunization Programs mapping with the WHO/Euro tool.
	*Leaflets developed in different languages* Germany: National level – information leaflets for each relevant vaccine in 20 languages. Bulgaria: Leaflets in the reception centres and refugee camps in Arabic, Farsi and other languages.
	*Poster and brochure distributed in camps regarding specific infectious disease* Poland: Information sessions were carried out in Centres for Foreigners (both for employees and for asylum seekers) about the importance of getting vaccinated and overall information on vaccine-preventable diseases. Brochures and posters regarding measles are distributed in the camps (prepared in cooperation with the National Institute of Public Health – National Institute of Hygiene).
Education and awareness	*Health education programmes* Distribution of educational material, developed by the International Organisation for Migration, in certain countries.
	*Information about vaccination distributed to migrants through the (registration) centre on arrival* Switzerland: Information on access to infectious diseases screening, access to care and access to vaccination is mandatory in centres for asylum seekers in federal registration centres and housing centres.
Outreach work	*Nurses visits and advice* Malta: Once migrants arrive, if they are undocumented and are in reception centres, nurses visit, advise and offer vaccines. Other migrants are reached through national immunisation campaigns.
	*Mobile outreach teams of physicians to migrant communities and reception centres* Reported in Germany.
	*Vaccination checking in school settings* Cyprus: “For children going to school, the school health services are very active in promoting the immunisations by checking all students for completeness of their vaccinations by asking them to present their immunisation cards. The parents of those students who don‘t have the necessary vaccines are contacted by phone by the school health visitor and they are asked to complete the missing vaccines for their child.”
National advocacy	*National immunisation campaigns* Reported in Malta.
	*Recommendations for vaccination promotion by health agencies and professionals* Austria: Targeted recommendations for vaccination upon first medical check, distributed to all involved stakeholders.
	*Governmental walk-in centres offer free vaccination for migrants* Cyprus: “People can get vaccinated or vaccinate their children in government walk-in centres completely free of charge. There are 63 such immunisation centres spread across Cyprus in cities and also in small communities.”

EEA: European Economic Area; EU: European Union; WHO/Euro: World Health Organization Regional Office for Europe.

### Recommendations to improve vaccination strategies in migrants

Most experts agreed that EU-level guidelines on the vaccination of migrants are needed (n = 26). Nineteen experts believed that vaccination should be better promoted. Other experts emphasised the need for detection of vaccine-preventable diseases (n = 6) and called for a new surveillance system to record information on vaccination status in asylum seekers. In addition, costs of vaccinations should be covered by national organisations (n = 4).

**Figure 2:**
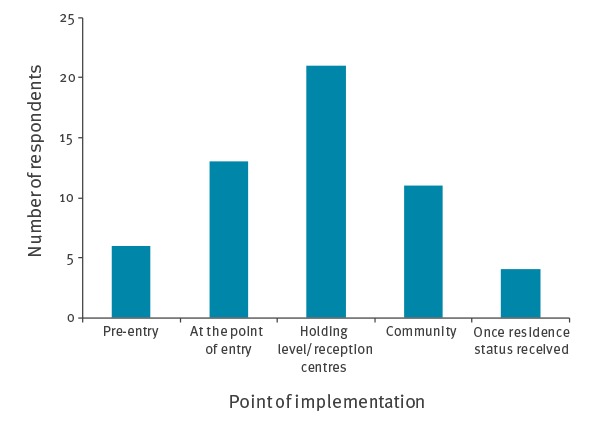
Vaccine experts’ opinion on when vaccinations should be offered to adult and child migrants, EU/EEA, 2017 (n = 32 countries)

To improve uptake of vaccination, experts highlighted that multiple opportunities for catch-up vaccination should be offered to adult and child migrants after arrival to the EU/EEA. Vaccination should be offered primarily at the point of entry or at a holding level (i.e. in reception centres, migrant camps and detention centres) (Figure 2).

## Discussion

Vaccination policies across Europe in relation to both adult and child migrants vary widely. Experts reported that national vaccination guidelines are used and these guidelines contain information on how to approach migrant patients with missed vaccinations and to offer catch-up vaccinations. However, these guidelines are often not migrant-specific and are frequently not applied in practice, particularly in relation to catch-up vaccination in adult migrants. Considerable variations in approaches exist between adults and children; children mostly enter the national vaccination schedule, whereas adults receive no catch-up vaccination or priority vaccinations only. The experts from 10 of 32 countries reported that migrants have to pay for vaccination. Almost half of the experts reported initiatives to promote vaccination among migrant groups, but evidence for a sound theoretical basis is lacking. Experts stated that better promotion for vaccination is needed and implementation should be strengthened. 

Priority is given to child vaccination in most EU/EEA countries. Vaccination guidelines include recommendations for children with incompletely vaccination. However, recommendations on the catch-up schedule to follow in case of missed vaccinations for both adults and children may vary by vaccine and by country. A study in 2011 that assessed immunisation policies across all EU/EEA countries – although not migrant-specific – found that all 27 countries recommended MMR and polio vaccinations for children, and only 11 of the 27 countries included MMR and polio vaccine for adults [24]. This concurs with our results pertaining to migrants, where vaccination for MMR and polio in migrant children is reported in all included countries and vaccination of adults only in half of the EU/EEA countries; this is despite the fact that some migrants originate from countries where health systems may have broken down resulting in immunisations being missed [25,26]. The Canadian guidelines, based on a systematic review of available evidence, recommend that MMR, DTP, polio, varicella and hepatitis B vaccines should be given to children and adult migrants [27], whereas our survey highlights that in more than half of EU/EEA countries these would not routinely be offered to adults and thus this represents an area requiring policy development [10]. Measles vaccine should be considered in both children and adult migrants in light of the fact that there have been outbreaks of measles in the EU that have been linked to migrant populations specifically, and there is a drive to eliminate measles in the European region [12,13]. Experts reported that hepatitis A was not routinely given, despite outbreaks of hepatitis being reported in migrant camps in Europe [11], yet the benefits of vaccinating migrants for hepatitis A may well be context-specific and something that needs to be considered a priority in camp and transit settings and/or focused on at-risk groups. EU/EEA countries may need to be mindful of additional vaccines such as influenza, hepatitis B and varicella vaccines that may need to be offered to migrants depending on living conditions, season and the epidemiological situation.

Barriers that have been shown to hinder adult vaccination uptake in general include lack of coordination, inability to pay and a lack of recommendation by healthcare providers [28,29], which echoes our findings pertaining to recently arrived migrants. Most migrants and refugees will not routinely be given a portable health record on arrival to the EU/EEA, so if they do get vaccinated in a transit or arrival country, this may result in duplication and/or confusion as to which vaccinations to give on arrival in the final destination country. The role of portable health records to ensure that a record is made of the vaccinations given at various points in the migration trajectory, with the aim of preventing duplication of vaccination, was not something we explored in this survey, but is an area that needs to be better considered. The International Organization for Migration (IOM), co-funded by the EU Third Health Programme, is currently piloting general health and vaccination assessments and exploring the role of electronic personal health records (e-PHR) in certain migrants arriving to Croatia, Greece, Italy and Slovenia as a tool for integration of refugees into EU health systems (https://greece.iom.int/en/re-health). To implement guidelines more effectively, the experts we approached recommended strong promotional campaigns and a harmonised vaccination schedule, which has been reported by others [5,30]. Educational activities to promote vaccination uptake by migrants were diverse, but the impact of these activities has not been well researched to date. These educational activities may benefit from further European collaboration, which has the potential to facilitate exchange of material in the appropriate languages and exchange of methods with measurable effects on vaccination uptake. The experts we approached called for EU-level guidelines to inform optimal approaches, which would support the goals of the European Vaccine Action Plan. Differences in countries’ healthcare systems and vaccine delivery structures need to be addressed [31]. Early access to primary care providers may be helpful in coordinating vaccine campaigns; yet it is unclear to what extent different countries have charging systems in primary care specifically for vaccination and this is not something we asked about in our survey. Another major challenge in terms of implementation is that countries across Europe face vastly different migrant situations – for example, transit countries such as Greece and Italy have large numbers of refugees arriving who may be temporary, which has implications for guideline development. The financing of vaccination programmes also needs to be considered in the context of migration. To improve uptake of vaccination in migrants, costs for key vaccinations should ideally be free of charge for migrants who are unable to pay, with European governments being mindful of their commitments to ensure equitable access to vaccines to meet target 3.8 of the Sustainable Development Goal on health to provide “access to safe, effective, quality, and affordable essential medicines and vaccines for all” [32]. Routine checks of vaccination status in medical files may facilitate the identification of missing vaccinations that can be addressed if migrants visit statutory healthcare providers after arrival. 

Clinicians and policymakers should also be mindful that both EU migrants – moving from one country in Europe to another – and non-EU migrants that were the focus of our survey, may be underimmunised and at increased risk for vaccine-preventable diseases; highly mobile EU migrants moving from eastern Europe to western Europe are a focus of the recent large multi-country measles epidemic in Europe [32]. In addition, more research is needed to explore catch-up vaccination in adolescent migrants who – alongside the adults identified in our survey – may also be an underimmunised group who are excluded from initiatives to assess immunisation status and offer appropriate catch-up vaccination, with national vaccination initiatives largely focused around children under 5 years of age [7,32]. In a cohort of asylum seekers in Denmark, 401 (48%) of 842 adolescents (aged 10–17 years) were reported as unimmunised or status unknown [7]. Further, it is important to note that migrants are one of several potentially underimmunised groups in the EU/EEA region. Data are lacking to what extent underimmunised groups contribute to outbreaks of vaccine-preventable diseases in the region, and improving data collection around migrant status and vaccine-preventable diseases, is an important next step. Table 2 summarises key points of action.

**Table 2:** Findings and points of action for governments, researchers and policy makers proposed by the vaccine expert survey, 2017 (n = 32 EU/EEA countries)

Findings	Suggested solutions
• There are a variety of approaches to vaccination of both adult and child migrants across the EU/EEA. • Where guidance exists, it is in most cases not migrant-specific and often not applied in practice. • Considerable variation in approaches exists between adults and children. Child migrants with uncertain vaccination status are in most countries re-vaccinated according to the national vaccination schedule. Adults often receive no catch-up vaccination, or for priority vaccinations only. • Adult migrants may be charged for vaccinations received at statutory health services in some countries, which may deter them from seeking vaccination and other preventative healthcare. • There is considerable variation among experts as to which vaccines should be offered to recently arrived migrants, particularly adults, and experts call for clear evidence-based guidance on this issue. • It is unclear where vaccination should be offered to improve uptake. Most experts agreed that focus should be soon after arrival, at the holding level (reception centres, refugee camps or detention centres) and be better promoted.	• Develop EU-level guidelines for vaccination of recently arrived adult and child migrants, with clarification given on which vaccines should be offered. • Multiple approaches are needed to engage and promote vaccination uptake in migrants, across multiple locations. • Vaccination needs to be free of charge for all migrant groups, including undocumented migrants. • Better explore models of best practice from across the EU/EEA to assess innovative strategies to improve vaccine delivery to adult and child migrants. • High quality studies are needed assessing vaccination implementation and cost-effectiveness in migrant populations. • Explore options for improving data collection and surveillance on vaccination coverage and burden of vaccine-preventable diseases in migrant populations across Europe. • Explore options for improving data capture to avoid duplication of efforts and unnecessary repeat vaccination along the migration trajectory (for example by non-governmental organisations in transit camps and also at statutory health services) after arrival to Europe (e.g. use of mobile phones, electronic vaccination cards and personal health records). • Explore the role of migrants (including underimmunised internal EU migrants and of adolescent and adult migrants), in outbreaks of vaccine-preventable diseases in the EU through robust research, and identify strategies to facilitate improved vaccine coverage in these groups.

EU/EEA: European Union/European Economic Area.

A limitation of our study is that we asked one expert at national level for each specific EU/EEA country and Switzerland, which may mean we have missed documenting regional differences. In addition, although we clearly defined at the top of the questionnaire the types of recently arrived migrants that we were aiming to capture data on, an expert’s own definition of a migrant may have meant that some answers did not fully represent the target group. Our experts were all working in the Ministry of Health or in public health, but we are aware that there are other entities providing vaccination, e.g. non-governmental organisations. We cannot conclude from our findings that the policies we have documented are applied in practice country-wide. A strength of our study is that we included all 32 EU/EEA countries and Switzerland, with a previous study exploring similar issues in six countries [33].

## Conclusion

Our data show that there is a need for migrant-specific guidelines on vaccination approaches for both children and adults in the EU/EEA. To improve uptake, guidelines on vaccination in migrant populations should include specific information on implementation. Further European collaboration has the potential to strengthen initiatives to improve vaccination uptake in underimmunised migrant groups.
